# Authors’ Reply: Foundation Models for Generative AI in Time-Series Forecasting

**DOI:** 10.2196/79772

**Published:** 2025-07-25

**Authors:** Rosemary He, Jeffrey Chiang

**Affiliations:** 1 Department of Computer Science University of California, Los Angeles Los Angeles, CA United States; 2 Department of Computational Medicine University of California, Los Angeles Los Angeles, CA United States; 3 Department of Neurosurgery David Geffen School of Medicine University of California, Los Angeles Los Angeles, CA United States

**Keywords:** generative artificial intelligence, artificial intelligence, time series, electronic health records, electronic medical records, systematic reviews, disease trajectory, machine learning, algorithms, forecasting

We thank the authors for their thoughtful letter [1] regarding our article “Generative AI Models in Time-Varying Biomedical Data: Scoping Review” [2]. We appreciate their careful reading and constructive feedback, which provides us with an opportunity to clarify important aspects of our work and address areas where our presentation may have been unclear.

We acknowledge the authors’ concern regarding our definition of foundation models (FMs). Their observation is well-taken, and we recognize that our use of terminology was imprecise in certain instances. We appreciate this opportunity to provide clarification. In our work, we intended to refer to FMs as models that have been trained on extremely large and typically unlabeled datasets, encompassing both models that are inherently capable of generative tasks and models that can be adapted for generative forecasting tasks (eg, clinical language models). This broader conceptualization reflects the evolving landscape of how these models are being applied in biomedical time-series analysis, where the distinction between inherently generative models and models adapted for generative purposes is becoming increasingly nuanced. However, the authors raise an important point about the distinction between masked language models and truly generative artificial intelligence (AI) models. We acknowledge that models like GatorTron are indeed masked language models rather than generative AI models in the strictest sense and appreciate the reference to GatorTronGPT as an example of a true generative variant.

The authors identified an important error in our presentation in Figure 3; we incorrectly labeled certain models as “foundation models” when they should have been categorized as “generative models.” While TimeGPT and denoising diffusion probabilistic models at large scale are FMs, the rest are generative models to be distinguished from FMs. We have corrected this label to avoid further confusion [3].

The field of generative AI applications to biomedical time-series data is rich and ever-growing, and the manuscript intends to offer multiple methodological options for researchers and practitioners. We thank the authors again for their careful attention to our work and their constructive feedback, which ultimately serves to strengthen the scientific discourse in this important area.

## Abbreviations

AI: artificial intelligence
FM: foundation model
